# Neglected Superior Ophthalmic Vein Enlargement before Delayed Symptom of Carotid-Cavernous Fistula in a Blowout Fracture: A Case Report and Literature Review

**DOI:** 10.1055/a-2258-2586

**Published:** 2024-06-14

**Authors:** Sunkyu Park, Inhoe Ku, Ji-Ung Park

**Affiliations:** 1 Department of Plastic and Reconstructive Surgery, Seoul National University Boramae Hospital, Seoul National University College of Medicine, Seoul, Republic of Korea

**Keywords:** carotid-cavernous fistula, superior ophthalmic vein, orbital wall fracture

## Abstract

Carotid-cavernous fistula (CCF) is a rare condition. However, it should be suspected when there are traumatic facial fractures, because if not diagnosed, it can lead to permanent damage such as blindness. Traumatic CCF often presents delayed symptoms, and delayed diagnosis without prompt treatment can lead to permanent injuries in optic and cranial nerves III, IV, V, and VI as well as intracranial hemorrhage. The routine initial modality for patients with suspected facial bone fractures is noncontrast computed tomography (CT) to identify any fracture lines and check for intracranial hemorrhage. We report a post-traumatic CCF case with a 4-day symptom delay, where left superior ophthalmic vein (SOV) enlargement was observed on the routine noncontrast facial CT with ipsilateral orbital wall fracture. When the patient first presented to the emergency room (ER), we did not detect vein enlargement on CT. Afterwards, the patient developed delayed symptoms of CCF and was readmitted to the ER. When we reanalyzed the first CT scan, an enlarged SOV was confirmed. The diagnosis was confirmed via magnetic resonance imaging angiography, and the patient was successfully treated with embolization of the fistula. Thus, we recommend reviewing ophthalmic vein enlargement that is readily identifiable through noncontrast CT for patients injured by craniofacial trauma to suspect the presence of delayed CCF at their initial presentation.

## Introduction

Carotid-cavernous fistula (CCF) is an abnormal communication between the cavernous sinus (CS) and carotid arterial system. 1 Although CCF occurs either spontaneously or by trauma, the most common etiology of CCF is craniofacial trauma, representing 69 to 79% of total CCF cases, and 0.17 to 0.27% of known craniofacial fractures are involved in CCF. 2 3 The carotid arterial system can be injured by shear stress caused by trauma or bone fragment penetration after sphenoid bone or skull base fractures. 4 CCF allows arterial high-pressure blood to flow into the CS and then back to the superior or inferior ophthalmic veins or superior or inferior petrosal sinus. This venous backflow causes a classic triad of symptoms, including pulsative proptosis, venular dilation with chemosis, and temporal or orbital bruit. Furthermore, cranial nerves II, III, IV, V, and VI, palsy, exophthalmos, glaucoma, visual acuity reduction, and headaches can be present in CCF. CCF symptoms and symptom onset periods vary with the nature of the fistula and hemodynamics. As the onset of extrinsic symptoms can be delayed, the diagnosis and treatment can also be delayed. Before these signs appear, the superior ophthalmic vein (SOV) can be engorged by backflow from high-pressure arterial flow and cause congestion around the venous draining tissues. Early detection of SOV enlargement is key to anticipate CCF signs and consider further workup to confirm CCF diagnosis. Herein, we report a case in which the patient presented with a blowout fracture and ipsilateral SOV enlargement on noncontrast computed tomography (CT), who was diagnosed with CCF 4 days later.

## Case

**Fig. 1:**
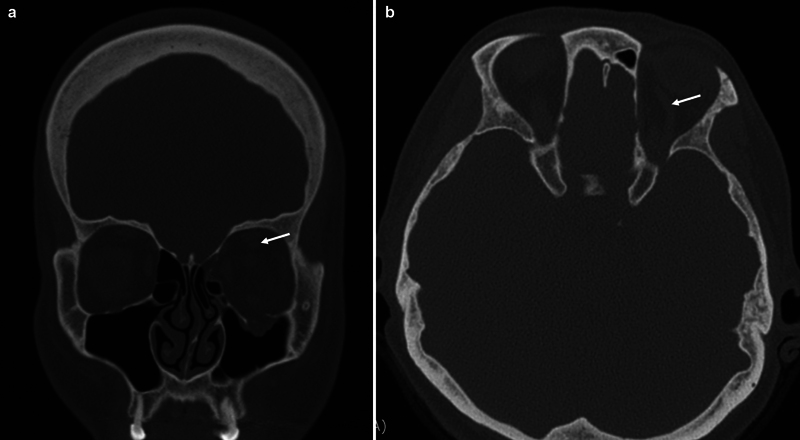
Noncontrast CT of the patient's first hospital visit. Superior ophthalmic vein dilatation is confirmed. ( **a** ) Coronal view. ( **b** ) Axial view. CT, computed tomography.

**Fig. 2:**
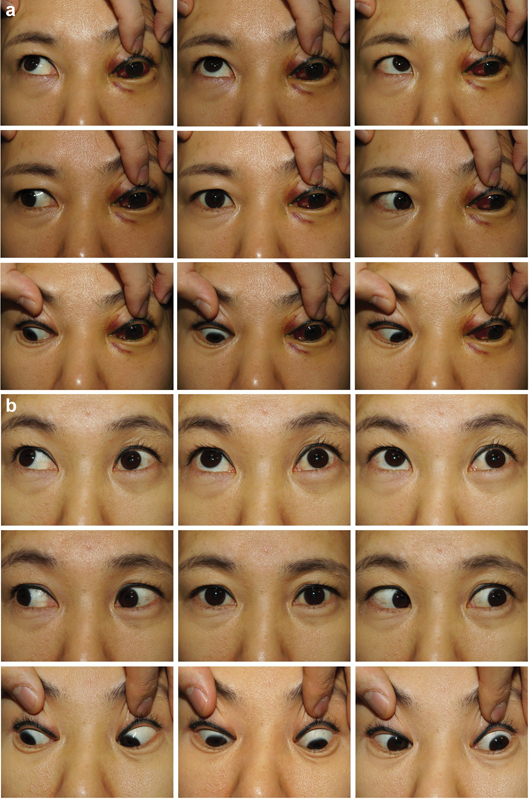
The ophthalmologic examination for the evaluation of function of the oculomotor nerve. ( **a** ) The left eye of the patient showed extraocular movement limitation, suspecting for oculomotor nerve palsy. ( **b** ) One month after embolization of CCF, the oculomotor nerve palsy was resolved, and the left eye showed almost normal extraocular movement. CCF, carotid-cavernous fistula.

**Fig. 3:**
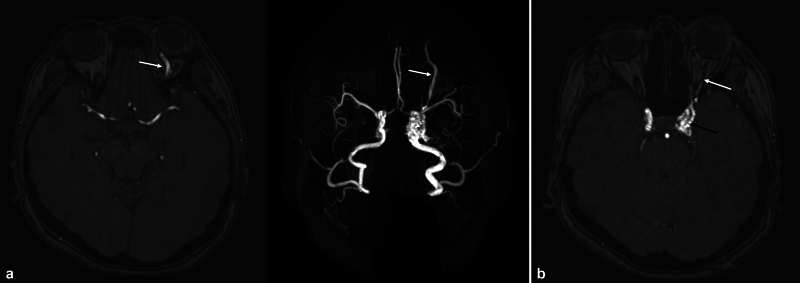
Brain MRA of the patient presenting with triad (pulsative proptosis, chemosis, and orbital bruit). ( **a** ) Superior orbital vein dilatation (arrow). ( **b** ) Prominent flow signal in the left cavernous sinus (black arrow) and oculomotor nerve enhancement (white arrow). MRA, magnetic resonance angiography.

A 44-year-old woman without underlying disease presented to the hospital with bruising of the left eye and numbness of the left cheek after her left eye was punched with a fist. Initial noncontrast-enhanced CT revealed a blowout fracture of the left orbit ( Fig. 1 ). At that time, we found no other remarkable findings, so we arranged for an outpatient clinic appointment and discharged the patient. Four days later, she presented to the emergency room with new-onset conjunctival injection, exophthalmos, orbital bruits, and pulsatile tinnitus in the left ear. Ophthalmological examination of the left eye showed eyelid ptosis and extraocular movement limitation, suggestive of pupil-sparing oculomotor nerve palsy ( Fig. 2a ). These symptoms were sufficient to suspect CCF, and we reanalyzed the CT scan taken during the first ER visit and confirmed that the SOV was enlarged ( Fig. 1 ). Brain magnetic resonance angiography (MRA) images showed a bulging carotid sinus wall and SOV enlargement, suggesting CCF and oculomotor nerve enhancement for neuropathy ( Fig. 3 ). Internal carotid angiography confirmed arterial flow drainage from the left carotid sinus to the ophthalmic vein and abundant collateral flow through the anterior communicating artery from the right internal carotid artery (ICA). Endovascular embolization subtotally occluded the fistula and the shunt to the SOV subsided. Immediately, postoperatively, pulsation of the left eyeball disappeared. The patient was discharged without complications. One month after the procedure, the oculomotor nerve palsy was resolved, and extraocular movements were almost fully restored ( Fig. 2b ). After approximately 6 months, the patient's extraocular movements were fully recovered.

## Discussion

A CCF is an abnormal connection between the ICA, ECA, or both, and the CS. The blood pressure in the carotid artery is much higher than that in the CS; therefore, if there is a communication between the two, it interferes with normal venous flow.

The CS is a dural venous sinus centered around the sella turcica and anatomical upper margin of the sphenoidal sinus. 4 The CS acts as a confluence of venous systems draining the orbit via the superior and inferior ophthalmic veins, anterior and middle cranial fossae via the sphenoparietal sinus, and posterior cranial fossa via the superior and inferior petrosal sinuses of the basilar plexus. 5 The cavernous segment of the ICA passes through the CS. The venous drainage pattern of the CS and the path of the ICA are associated with the pathophysiology of CCF.

CCF can be classified into direct and indirect. A direct connection between the ICA and the CS can be classified as a direct CCF, whereas indirect CCFs are characterized by the connection between the ICA branches or the external carotid artery (ECA) and the CS. Direct CCF is frequently caused by craniofacial trauma, ICA aneurysm rupture, or iatrogenic interventions. This direct CCF causes rapid blood backflow from the CS into the SOV, leading to SOV dilation and ophthalmic symptoms. In view of the above, this case can also be classified as a direct CCF.

Indirect CCF are mostly low-flow fistulae that are caused by the connection between the meningeal branches of the ICA and/or ECA and the CS. The most indirect CCF occurs spontaneously and mostly as a degenerative change with hypertension and/or atherosclerosis. 6

The clinical presentation and symptom onset period can vary depending on the classification, size, and etiology. Early clinical features generally include the orbit due to the venous drainage route from the ophthalmic vein to the CS. CCF often shows a triad of pulsative proptosis, venular dilation with chemosis, and temporal or orbital bruit. Patients may experience diplopia, chemosis, orbital pain, tinnitus, headaches, or vision loss. The symptoms are mostly ipsilateral to the fistula but can occur on both sides. 7 Cranial nerve deficits due to ischemic changes in cranial nerves II, III, IV, V, and/or VI, can occur and change over time.

A detailed history and clinical examination with appropriate diagnostic tests can be helpful for identifying and classifying CCF. Most patients undergo noninvasive imaging with CT, magnetic resonance imaging, CT angiography, or MRA. The common manifestations of CCF on noninvasive imaging are proptosis and dilation of the SOV, CS, and extraocular muscle enlargement. 8 However, in patients with CCF, not only can the dilation of the SOV be observed, but internal thrombosis may also be detected, which can be accompanied by symptoms such as periorbital pain and congestion, and is well-visualized on CT imaging with contrast agent. 9 10 If there is evidence of CCF on noninvasive imaging, diagnostic cerebral angiography can confirm the presence of CCF and guide treatment.

When a patient has a history of craniofacial trauma, the first imaging modality is noncontrast enhanced CT, which is noninvasive, to rule out intracranial bleeding and craniofacial fracture. Superior ophthalmic dilation or thrombosis, extraocular muscle thickening, and periorbital fat edema, which are identifiable on CT scans, can indicate CCF. 11 Although noncontrast use for SOV identification may be less apparent than when contrast is used, signs of SOV ectasia in craniofacial trauma patients can still be observed on noncontrast CT, as in the present case. Therefore, we advocate reviewing SOV enlargement on the initial noncontrast-enhanced CT to inspect traumatic CCF.

When SOV enlargement is identified using noncontrast CT, CT angiography or MRA is recommended to detect CCF with high sensitivity. Thus, minimal SOV dilation, proptosis, and extraocular muscle thickening can be more readily identified. 11 In the presence of these clinical signs on noncontrast CT, the possibility of CCF must always be considered, and further evaluation should be performed.

However, there is a chance that the fistula drains through the petrosal sinus and intracranial venous channel, curtails the flow into the ophthalmic veins, and obscures ophthalmic vein dilatation. In such cases, cortical venous drainage may occur, which can lead to intracranial hemorrhage. 12

When CCF is diagnosed, treatment must proceed, and the treatment strategy depends on the etiology, clinical manifestations, and risk of neurological and ophthalmic complications. Patients with mild ocular symptoms can be treated with topical medications and observed during routine follow-ups to assess visual acuity, ophthalmoscopic changes, and intraocular pressure. Direct CCF is mostly symptomatic and requires urgent treatment. Endovascular treatment is the first-line therapy with an approach to closing the fistula.

If effective intervention is performed for CCF resulting in fistula closure, symptoms such as the triad of pulsatile proptosis, venular dilation with chemosis, and temporal or orbital bruit, which are characteristic of CCF, are immediately alleviated. 13 Cranial nerve deficits generally show recovery progress over several weeks. 14 15 In the case of visual impairment, the extent of visual damage and the time elapsed between the occurrence of visual impairment and intervention can influence the degree of recovery. Because it can lead to blindness, the possibility of CCF should always be kept in mind in patients with facial trauma. 1 16 17

### Conclusion

CCF is a rare but it can be an encountered condition in patients with facial bone fractures and if missed, it can lead to fatal results. However, symptoms of CCF often develop late; thus, the diagnosis is often delayed. Permanent cranial nerve injuries may occur when appropriate treatment is delayed. We would like to introduce the possibility of early detection of the CCF sign on noncontrast facial bone CT, which is routinely performed to confirm fractures in patients with craniofacial trauma. In this case, SOV dilation was confirmed on noncontrast CT, and the possibility of CCF should have been known. However, later, after the patient developed symptoms, we suspected CCF and treatment was initiated at that time. Fortunately, the patient recovered without complications, but if there is no timely intervention , permanent damage could have occurred. Therefore, when trauma that can damage the structure around the carotid-cavernous, such as orbital wall fractures, is observed, the possibility of CCF can be considered by checking the expansion of the superior or inferior optical valve with initial noncontrast CT.

## References

[1] Chaudhry I A Elkhamry S M Al-Rashed W Bosley T M Carotid cavernous fistula: ophthalmological implications Middle East Afr J Ophthalmol 2009 16 02 57 63 20142962 10.4103/0974-9233.53862 PMC2813585

[2] Millman B Giddings N A Traumatic carotid-cavernous sinus fistula with delayed epistaxis Ear Nose Throat J 1994 73 06 408 411 8076541

[3] Nocini P Lo Muzio L Cortelazzi R Barbaglio A Cavernous sinus-carotid fistula: a complication of maxillofacial injury Int J Oral Maxillofac Surg 1995 24 04 276 278 7490489 10.1016/s0901-5027(95)80028-x

[4] de Moraes S L de Paula Afonso A M Dos Santos R G Mattos R P Duarte B G Carotid-cavernous fistula as a complication of panfacial fracture: Case report 11 years after the surgery Craniomaxillofac Trauma Reconstr 2017 10 01 66 72 28210411 10.1055/s-0036-1582458 PMC5305314

[5] Rhoton A L Jr The cavernous sinus, the cavernous venous plexus, and the carotid collar Neurosurgery 2002 51(4 Suppl)S375 S410 12234454

[6] Yu S S Lee S H Shin H W Cho P D Traumatic carotid-cavernous sinus fistula in a patient with facial bone fractures Arch Plast Surg 2015 42 06 791 793 26618131 10.5999/aps.2015.42.6.791 PMC4659997

[7] Lerut B De Vuyst C Ghekiere J Vanopdenbosch L Kuhweide R Post-traumatic pulsatile tinnitus: the hallmark of a direct carotico-cavernous fistula J Laryngol Otol 2007 121 11 1103 1107 17295936 10.1017/S0022215107005890

[8] Miller N R Diagnosis and management of dural carotid-cavernous sinus fistulas Neurosurg Focus 2007 23 05 E13 10.3171/FOC-07/11/E13 18004961

[9] Sotoudeh H Shafaat O Aboueldahab N Vaphiades M Sotoudeh E Bernstock J Superior ophthalmic vein thrombosis: What radiologist and clinician must know? Eur J Radiol Open 2019 6 06 258 264 31641683 10.1016/j.ejro.2019.07.002 PMC6796573

[10] Michaelides M Aclimandos W Bilateral superior ophthalmic vein thrombosis in a young woman Acta Ophthalmol Scand 2003 81 01 88 90 12631033 10.1034/j.1600-0420.2003.00028_9.x

[11] Dos Santos D Monsignore L M Nakiri G S Cruz A A Colli B O Abud D G Imaging diagnosis of dural and direct cavernous carotid fistulae Radiol Bras 2014 47 04 251 255 25741093 10.1590/0100-3984.2013.1799 PMC4337128

[12] Vadivelu S Bell R S Crandall B DeGraba T Armonda R A Delayed detection of carotid-cavernous fistulas associated with wartime blast-induced craniofacial trauma Neurosurg Focus 2010 28 05 E6 10.3171/2010.2.FOCUS09257 20568946

[13] Ellis J A Goldstein H Connolly E S Jr Meyers P M Carotid-cavernous fistulas Neurosurg Focus 2012 32 05 E9 10.3171/2012.2.FOCUS1223 22537135

[14] Kirsch M Henkes H Liebig T Endovascular management of dural carotid-cavernous sinus fistulas in 141 patients Neuroradiology 2006 48 07 486 490 16639562 10.1007/s00234-006-0089-9

[15] Wang W Li Y D Li M H Endovascular treatment of post-traumatic direct carotid-cavernous fistulas: A single-center experience J Clin Neurosci 2011 18 01 24 28 20888773 10.1016/j.jocn.2010.06.008

[16] Liang W Xiaofeng Y Weiguo L Wusi Q Gang S Xuesheng Z Traumatic carotid cavernous fistula accompanying basilar skull fracture: a study on the incidence of traumatic carotid cavernous fistula in the patients with basilar skull fracture and the prognostic analysis about traumatic carotid cavernous fistula J Trauma 2007 63 05 1014 1020, discussion 1020 17993945 10.1097/TA.0b013e318154c9fb

[17] Kim M S Han D H Kwon O K Oh C W Han M H Clinical characteristics of dural arteriovenous fistula J Clin Neurosci 2002 9 02 147 155 11922702 10.1054/jocn.2001.1029

